# Liquid biopsy-based genomic risk score to predict neurologic death in non-small cell lung cancer patients

**DOI:** 10.1007/s11060-026-05642-z

**Published:** 2026-05-25

**Authors:** Sarah E. Glynn, Ralph D’Agostino Jr, Claire M. Lanier, Ariel R. Choi, Michael Farris, Mohammed Abdulhaleem, Patrick Young, Yuezhu Wang, Margaret Smith, Jimmy Ruiz, Thomas Lycan, William Jeffrey Petty, Christina K. Cramer, Stephen B. Tatter, Adrian W. Laxton, Jaclyn J. White, Jing Su, Christopher T. Whitlow, Fei Xing, Michael D. Chan, Corbin A. Helis

**Affiliations:** 1 Department of Radiation Oncology, Wake Forest University School of Medicine, Winston Salem, NC USA; 2 Department of Biostatistics and Data Science, Wake Forest University School of Medicine, Winston Salem, NC USA; 3 Department of Hospital Medicine, Wake Forest University School of Medicine, Winston Salem, NC USA; 4 Department of Molecular and Cellular Bioscience, Wake Forest University School of Medicine, Winston Salem, NC USA; 5 Department of Internal Medicine, Section of Hematology and Oncology, Wake Forest University School of Medicine, Winston Salem, NC USA; 6 Department of Neurosurgery, Wake Forest University School of Medicine, Winston Salem, NC USA; 7 Department of Biostatistics and Health Data Science, Indiana University School of Medicine, Indianapolis, IN USA; 8 Department of Diagnostic Radiology, Wake Forest University School of Medicine, Winston Salem, NC USA; 9 Department of Cancer Biology, Wake Forest University School of Medicine, Winston Salem, NC USA

**Keywords:** Brain metastases, Next generation sequencing, Non-small cell lung cancer, Genetic biomarkers, Radiotherapy, Brain death

## Abstract

**Introduction:**

Biomarkers that predict neurologic death may allow for personalization of therapy for high-risk brain metastases patients.

**Methods:**

Patients with NSCLC who underwent comprehensive genomic profiling were identified in an institutional database. Neurologic death was determined by medical record review. Proportional hazards regression models considering non-neurologic death as a competing risk were used to identify mutations statistically associated with the occurrence of neurologic death (*p* < 0.1) and to create a risk scoring system for neurologic death. A competing risk proportional hazards regression model with non-neurologic death as a competing risk was used to assess the association between the risk score and neurologic death, and to calculate hazard ratios predicting neurologic death between risk groups.

**Results:**

307 patients were included in the primary analysis and 213 in a cohort of patients with brain metastases. Risk scores were constructed in both populations. Patients with higher risk scores had an increased risk of neurologic death when compared to those in the low-risk group, with respective HRs of 3.76 for the entire cohort and 2.87 for the brain metastasis cohort per unit increase in the risk score. When dividing the risk score into three groups, the cumulative incidence of neurologic death in high, moderate, and low risk groups was 49.0%, 20.3% and 0% for the entire cohort and 52.4%, 30.4%, and 0% in the brain metastasis cohort.

**Conclusion:**

Development of a genomic signature to predict neurologic death via non-invasive liquid biopsy appears feasible in NSCLC patients.

## Introduction

Approximately 200,000 patients in the United States are diagnosed with brain metastases each year [1]. In the past, the diagnosis of brain metastasis led to a high mortality rate due to the likelihood of death from neurologic causes [2]. However, over the past two decades, there has been a stage migration from large metastases diagnosed because of symptomatic presentation to a population with small asymptomatic metastases discovered during staging protocols [3]. Because of this change in the characteristics of the brain metastasis population, there has been a shift in whether metastatic cancer patients die of brain metastases or from other causes, such as their primary cancer, distant metastases, or intercurrent disease [4].

The decrease in neurologic death is generally due to lead-time bias from earlier diagnosis, though novel systemic therapies [5, 6] and CNS-directed therapies [7, 8] have also contributed to this improvement in outcomes. Despite improvements and shifts in the brain metastasis population, a significant proportion of patients with brain metastases will still die from neurologic causes. In the modern population, recent trials have shown that nearly 20% of patients with brain metastasis will experience neurologic death [9]. Potential risk factors for higher neurologic death rates include melanoma histology [10], high brain metastasis velocity [11], brainstem location [12] and larger tumors [8].

Recent analyses have demonstrated that genomic profiling can be used to predict brain metastasis behavior, including which patients may develop brain metastases [13] and which patients develop larger or more numerous metastases [14]. As such, it was hypothesized that genetic profiling might also predict the risk of brain metastasis patients dying of neurologic death. As this remains a concern in the modern brain metastasis population, several options could be considered for patients who are at increased risk of neurologic death, including surveillance, treatment intensification, and prophylaxis.

The present study represents a retrospective analysis of a population of patients with metastatic non-small cell lung cancer who underwent comprehensive genomic profiling with a proportion developing brain metastases at some point during their natural history. We hypothesized that we could use genomic profiling to classify patients into distinct risk groupings that were predictive of time to neurologic death, which could have downstream clinical utility such as escalating upfront treatment and increasing surveillance.

## Methods

### Data acquisition

This study was approved by the Wake Forest School of Medicine Institutional Review Board, IRB00033881. Patients with non-small cell lung cancer were identified from our prospective institutional database of patients with comprehensive genomic profiling. Patients were included in this study if their comprehensive genomic profiling (CGP) was done using the Guardant platform. Patients with CNS metastases at time of diagnosis, those who developed CNS metastases during their lives and those who never developed CNS metastases were all included in the study to determine if genomic factors could differentiate between positive and negative outcomes regardless of initial clinical staging. The electronic medical record (EMR) was then used to determine which of these patients developed brain metastases during their natural history and to identify patients who died of neurologic death, as defined in the publication by McTyre et al. [15] and Patchell et al. [16]. In brief, cause of death was considered neurologic death if a patient died with progressive neurologic deterioration or severe neurologic dysfunction, typically characterized by an uncontrolled increase in intracranial tumor burden, irrespective of their extracranial disease status.

### Comprehensive genomic profiling

Comprehensive genomic profiling (CGP) was performed using the Guardant 360 platform (Guardant Health, Palo Alto, CA). Test samples were acquired via liquid biopsy performed on peripheral blood, which were obtained in accordance with National Comprehensive Cancer Network (NCCN) guidelines prior to the administration of systemic therapy, as previously published by Leighl et al.[17] This test uses circulating tumor DNA in patient samples to assess for the presence of known mutations common to NSCLC, including *AKT1*,* ALK*,* APC*,* AR*,* ARAF*,* ARID1A*,* ATM*,* BRAF*,* BRCA1*,* BRCA2*,* CCND1*,* CCNE1*,* CDH1*,* CDK4*,* CDK6*,* CDK12*,* CDKN2A*,* CHEK2*,* CTNNB1*,* DDR2*,* EGFR*,* ERBB2*,* ESR1*,* EZH2*,* FANCA*,* FBXW7*,* FGFR1*,* FGFR2*,* FGFR3*,* GATA3*,* GNA11*,* GNAQ*,* GNAS*,* HNF1A*,* HRAS*,* IDH1*,* IDH2*,* JAK2*,* JAK3*,* KEAP1*,* KIT*,* KRAS*,* MAP2K1*,* MAP2K2*,* MAPK1*,* MET*,* MLH1*,* MPL*,* MSH2*,* MSH6*,* MTOR*,* MYC*,* NF1*,* NFE2L2*,* NOTCH1*,* NPM1*,* NRAS*,* NTRK1*,* NTRK2*,* NTRK3*,* PALB2*,* PDGFRA*,* PIK3CA*,* PMS2*,* PTEN*,* PTPN11*,* RAD51D*,* RAF1*,* RB1*,* RET*,* ROS1*,* SMAD4*,* SMO*,* STK11*,* TERT*,* TP53*,* TSC1* and *VHL*.

### Statistics

In order to create a risk stratification score for the prediction of neurologic death, a series of Cox proportional hazards regression models were fit to identify genetic mutations associated with time to neurologic death in the full cohort (*n* = 307) of patients. A *p*-value of < 0.1 was used for this first screening of statistical association. Genes with statistical associations were assigned a score of + 1 if they led to an increased risk of neurologic death (i.e. had a hazard ratio greater than 1 for predicting time to neurologic death), or -1 if they led to a decreased risk of neurologic death (i.e. hazard ratio less than 1). The sum of these values was calculated to define a genetic risk score (with scores ranging from negative to positive values) and a 3-level risk score (high, moderate, and low risk), with respective scores of > 0, 0, and < 0. A competing risk proportional hazards regression model with non-neurologic death as a competing risk was then used to assess the association between the genetic risk score and neurologic death, and to calculate hazard ratios (HR) predicting neurologic death for each unit increase of the genetic risk score. We additionally examined overall survival, using the 3-level genetic risk score and the overall genetic risk score, and compared groups using Log-Rank tests.

Additional exploratory and sensitivity analyses were conducted to evaluate the clinical significance of the genetic risk score. The association between overall survival and genetic risk score was assessed using a log-rank test comparing Kaplan-Meier curves across the three risk score groups. Next, the association of the genetic risk score with neurologic death was examined among subgroups both with and without targetable mutations (*EGFR*, *ROS1*, *BRAF*, *NTRK3* and *ALK*), using competing risks regression. Finally, we constructed a multivariable competing risks proportional hazards model to investigate whether the genetic risk score remained independently associated with time to neurologic death when accounting for patient-level factors, including Karnofsky performance status (KPS), number of brain metastases, overall disease burden (none, oligometastatic, or widespread), and type of brain irradiation, including whole brain radiotherapy (WBRT), Gamma Knife radiosurgery (GKRS), both, or none.

## Results

### Patient population

A total of 307 patients underwent CGP between 2005 and 2021 and were included in the analysis. The median follow-up was 32.9 months. Of these 307 patients, 213 (69.4%) developed brain metastases over the course of the study, with 127 (41.4%) presenting with CNS metastases at time of initial diagnosis. A total of 246 of 307 patients in the entire cohort and 182 of 213 in the brain metastasis cohort had died at the time of the analysis. Neurologic death occurred in 63 patients: 21% of the entire population and in 29.6% of patients who developed brain metastases. Patient characteristics for all subjects, and for those in the neurologic death and non-neurologic death cohorts are summarized in Table 1. Patients experiencing neurologic death were younger and more likely to have advanced stage disease. At initial diagnosis, these patients had 4.7 versus 1.9 brain metastases, respectively (*p* = 0.001), and were more likely to receive brain-directed radiotherapy during their disease course. Patient characteristics among those who developed brain metastases are summarized in Table 2. Across the subset of patients who developed brain metastases, patients with *KRAS* mutations were more likely and those with *EGFR* mutations were less likely to be in the high-risk groups. There were no differences in the rates of *ALK*, *ROS1*, or *BRAF* mutations across the different risk groups. Additionally, there were no statistically significant differences in KPS, stage at diagnosis, or NSCLC histology in the brain metastasis subset.

**Table 1:** Patient characteristics of entire cohort, neurologic death cohort, and non-neurologic death cohort

	Entire Cohort n (%) / median (IQR)	Neurologic Death Cohort n (%) / median (IQR) (n=63)	Non-Neurologic Death Cohort n (%) / median (IQR) (n=244)	p-value
Age	67.4	65.1	68	0.024
Sex				0.048
Male	151	24 (15.9%)	127 (84.1%)	
Female	156	39 (25.0%)	117 (75.0%)	
Ethnicity				
White	266	52 (19.5%)	214 (80.5%)	0.538
African American/Black	38	10 (26.3%)	28 (73.7%)	
Other	3	1 (33.3%)	2 (66.7%)	
KPS				0.750
100	5	0 (0.0%)	5 (100%)	
90	61	9 (14.8%)	52 (85.2%)	
80	131	31 (23.7%)	100 (76.3%)	
70	59	17 (28.8%)	42 (71.2%)	
60	29	5 (17.2%)	24 (82.8%)	
50	10	1 (10.0%)	9 (90.0%)	
40	9	0 (0.0%)	9 (100%)	
Unknown	2			
Actionable Mutations				
EGFR				0.486
Yes	68	16 (23.5%)	52 (76.5%)	
No	239	47 (19.7%)	192 (80.3%)	
KRAS				0.778
Yes	93	20 (21.5%)	73 (78.5%)	
No	214	43 (20.1%)	171 (79.9%)	
ALK				0.676
Yes	18	3 (16.7%)	15 (83.3%)	
No	289	60 (20.8%)	229 (79.2%)	
ROS1				0.286
Yes	12	1 (8.3%)	11 (91.7%)	
No	295	62 (21.0%)	233 (79.0%)	
BRAF				0.559
Yes	25	4 (16%)	21 (84%)	
No	282	59 (20.9%)	223 (79.1%)	
Smoking Status				0.921
Former	191	39 (20.4%)	152 (79.6%)	
Current	67	13 (19.4%)	54 (80.6%)	
Never	49	11 (22.4%)	38 (77.6%)	
Stage				0.032
I	5	0 (0.0%)	5 (100%)	
II	4	0 (0.0%)	4 (100%)	
III	37	2 (5.4%)	35 (94.6%)	
IV	261	61 (23.4%)	200 (76.6%)	
NSCLC Histology				0.746
Adenocarcinoma	226	49 (21.7%)	177 (78.3%)	
Squamous cell carcinoma	47	9 (19.1%)	38 (80.9%)	
Large cell carcinoma	2	0 (0.0%)	2 (100%)	
NSCLC-NOS	32	5 (15.6%)	27 (84.4%)	
Diagnosed with Brain Metastases				<0.001
Yes	213	63 (29.6%)	150 (70.4%)	
No	94	0 (0.0%)	94 (100%)	
Number of brain metastases at initial diagnosis	2.5	4.7	1.9	0.001
Radiation				<0.001
None	115	7 (6.1%)	108 (93.9%)	
GK SRS	145	35 (24.1%)	110 (75.9%)	
WBRT	26	13 (50%)	13 (50%)	
GK SRS+WBRT	21	8 (38.1%)	13 (61.9%)	

**Table 2:** Characteristics of patients diagnosed with brain metastases

	Brain Metastasis Cohort n=213 n (%) / median (IQR)	High Risk n=42	Moderate Risk n=135	Low Risk n=36	p-value
		n (%) / median (IQR)	n (%) / median (IQR)	n (%) / median (IQR)	
Age	67.0 (60.0-73.0)	66.0 (56.0-73.0)	67.0 (61.0-72.0)	67.5 (60.5-74.5)	0.39
Sex					0.632
Male	100 (47.0%)	17 (17.0%)	65 (65.0%)	18 (18.0%)	
Female	113 (53.1%)	25 (25.1%)	70 (62.0%)	18 (15.9%)	
Ethnicity					0.636
White	185 (86.9%)	37 (20.0%)	115 (62.2%)	33 (17.8%)	
African American/Black	26 (12.2%)	4 (15.38%)	19 (73.1%)	3 (11.5%)	
Other	2 (0.9%)	1 (50.0%)	1 (50.0%)	0 (0.0%)	
KPS					0.77
100	3 (1.4%)	0 (0.0%)	2 (66.7%)	1 (33.3%)	
90	47 (22.2%)	9 (19.2%)	28 (59.6%)	10 (21.3%)	
80	92 (43.4%)	16 (17.4%)	61 (66.3%)	15 (16.3%)	
70	51 (24.1%)	12 (23.5%)	31 (60.8%)	8 (15.7%)	
60	14 (6.6%)	5 (35.7%)	8 (57.1%)	1 (7.1%)	
50	3 (1.4%)	0 (0.0%)	3 (100%)	0 (0.0%)	
40	2 (0.9%)	0 (0.0%)	2 (100%)	0 (0.0%)	
Actionable Mutations					
EGFR					0.024
Yes	49 (23.0%)	3 (6.1%)	36 (73.5%)	10 (20.4%)	
No	164 (77.0%)	39 (23.8%)	99 (60.4%)	26 (15.9%)	
KRAS					0.022
Yes	66 (31.0%)	20 (30.3%)	34 (51.5%)	12 (18.2%)	
No	147 (69.0%)	22 (15.0%)	101 (68.7%)	24 (16.3%)	
ALK					0.654
Yes	13 (6.1%)	3 (23.1%)	9 (69.2%)	1 (7.7%)	
No	200 (93.9%)	39 (19.5%)	126 (63.0%)	35 (17.5%)	
ROS1					0.223
Yes	9 (4.2%)	0 (0.0%)	8 (88.9%)	1 (11.1%)	
No	204 (95.8%)	42 (20.6%)	127 (62.5%)	35 (17.2%)	
BRAF					0.576
Yes	20 (9.4%)	4 (20.0%)	11 (55.0%)	5 (25.0%)	
No	193 (90.6%)	38 (19.7%)	124 (64.3%)	31 (16.1%)	
Smoking Status					0.662
Former	131 (61.5%)	26 (19.9%)	84 (64.1%)	21 (16.0%)	
Current	50 (23.5%)	11 (22.0%)	28 (56.0%)	11 (22.0%)	
Never	32 (15.0%)	5 (15.6%)	23 (71.9%)	4 (12.5%)	
Stage					0.756
I	2 (0.9%)	0 (0.0%)	2 (100.0%)	0 (0.0%)	
II	0 (0.0%)	0 (0.0%)	0 (0.0%)	0 (0.0%)	
III	7 (3.3%)	1 (14.3%)	4 (57.1%)	2 (28.6%)	
IV	204 (95.8%)	41 (20.1%)	129 (63.2%)	34 (16.7%)	
NSCLC Histology					0.869
Adenocarcinoma	166 (77.9%)	35 (21.1%)	103 (62.1%)	28 (16.9%)	
Squamous cell carcinoma	26 (12.2%)	3 (11.5%)	19 (69.2%)	5 (19.2%)	
Large cell carcinoma	2 (0.9%)	0 (0.0%)	2 (100.0%)	0 (0.0%)	
NSCLC-NOS	19 (8.9%)	4 (21.1%)	4 (21.1%)	3 (15.8%)	

### Genes associated with neurologic death

Genetic mutations in the NGS panel found to be associated with neurologic death in the entire cohort and in the subset of patients who developed brain metastases are detailed in Table 3. Deleterious genes common across both cohorts were *CDK4*, *PALB2*, and *GNAQ*. Protective genes in common across both cohorts were *CDK12*, *JAK2*, *TERT*, *CCND1*, *CHEK2*, *EZH2*, *HRAS*, *IDH2*, *JAK3*, *MAPK1*, *ARAF*, *IDH1*, *NPM1*, *GNA11*, *MPL*, and *MAP2K1*. For the entire cohort, an additional protective gene, *VHL*, was identified and two additional deleterious genes, *STK11* and *RAF1*, were identified. For the subset of patients who developed brain metastases, two additional deleterious genes, *PTPN11* and *NTRK3*, were identified.

**Table 3:** Genes associated with neurologic death

	Entire Cohort			Brain Metastases Patients		
*Gene*	Incidence *n* (%)	Hazard Ratio	*P*-Value	Incidence *n* (%)	Hazard Ratio	*P*-Value
*CDK4*	10 (3.26%)	3.569	0.0008	9 (4.23%)	712	0.007
*PALB2*	5 (1.63%)	4.219	0.015	5 (2.35%)	2.776	0.087
*GNAQ*	3 (0.98%)	5.238	0.025	3 (1.41%)	3.465	0.097
*STK11*	32 (10.42%)	1.965	0.042	N/A	N/A	N/A
*RAF1*	7 (2.28%)	2.855	0.093	N/A	N/A	N/A
*PTPN11*	N/A	N/A	N/A	1 (0.47%)	3.073	< 0.001
*NTRK3*	N/A	N/A	N/A	13 (6.1%)	2.145	0.087
*CDK12*	9 (2.93%)	< 0.001	< 0.001	3 (1.41%)	< 0.001	< 0.001
*JAK2*	10 (3.26%)	< 0.001	< 0.001	8 (3.76%)	< 0.001	< 0.001
*TERT*	13 (4.23%)	< 0.001	< 0.001	7 (3.29%)	< 0.001	< 0.001
*CCND1*	8 (2.61%)	< 0.001	< 0.001	6 (2.82%)	< 0.001	< 0.001
*CHEK2*	7 (2.28%)	< 0.001	< 0.001	6 (2.82%)	< 0.001	< 0.001
*EZH2*	7 (2.28%)	< 0.001	< 0.001	6 (2.82%)	< 0.001	< 0.001
*HRAS*	5 (1.63%)	< 0.001	< 0.001	2 (0.94%)	< 0.001	< 0.001
*IDH2*	4 (1.3%)	< 0.001	< 0.001	4 (1.88%)	< 0.001	< 0.001
*JAK3*	3 (0.98%)	< 0.001	< 0.001	1 (0.47%)	< 0.001	< 0.001
*MAPK1*	2 (0.65%)	< 0.001	< 0.001	1 (0.47%)	< 0.001	< 0.001
*ARAF*	2 (0.65%)	< 0.001	< 0.001	1 (0.47%)	< 0.001	< 0.001
*IDH1*	2 (0.65%)	< 0.001	< 0.001	1 (0.47%)	< 0.001	< 0.001
*VHL*	1 (0.33%)	< 0.001	< 0.001	1 (0.47%)	N/A	N/A
*NPM1*	1 (0.33%)	< 0.001	< 0.001	1 (0.47%)	< 0.001	< 0.001
*GNA11*	1 (0.33%)	< 0.001	< 0.001	1 (0.47%)	< 0.001	< 0.001
*MPL*	1 (0.33%)	< 0.001	< 0.001	1 (0.47%)	< 0.001	< 0.001
*MAP2K1*	1 (0.33%)	< 0.001	< 0.001	1 (0.47%)	< 0.001	< 0.001

### Risk stratification scores for neurologic death

**Fig. 1:**
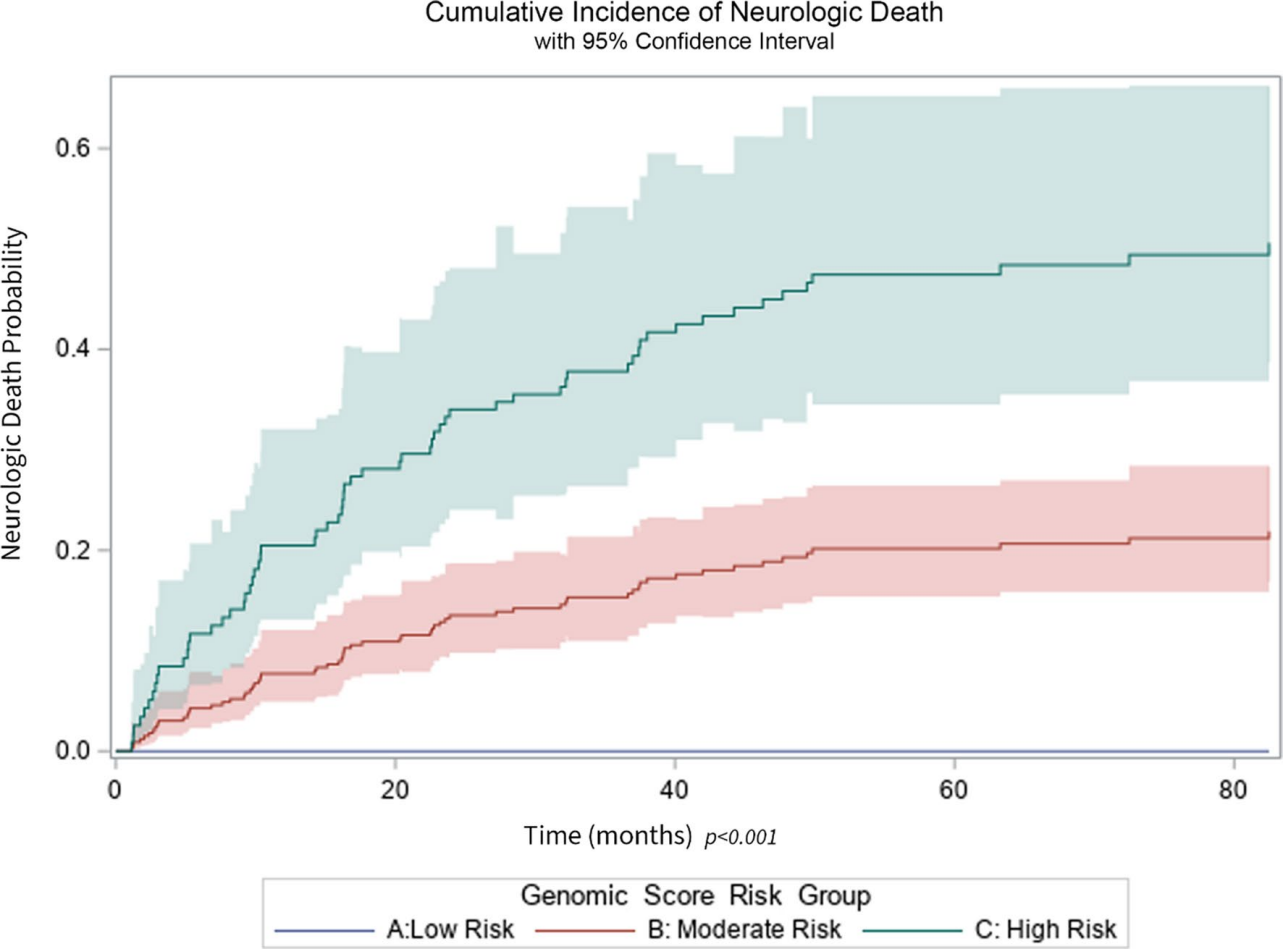
Probability of neurologic death among all patients by genomic score risk groups, low risk (blue), moderate risk (red) and high risk (green) *p* < 0.001

The genomic risk score that was constructed using the 22 genes described above had a score that ranged from − 3 to + 2, and this score was significantly associated with the risk of neurologic death with a HR of 3.76 (95% CI 2.72–5.18, *p* < 0.001), in the competing risk analysis using non-neurologic death as a competing risk. When the genomic risk score was categorized into three groups: high (risk score > 0), moderate (risk score = 0) and low (risk score < 0), we found that 45, 202, and 60 participants fell into these three categories, respectively. When comparing these three risk groups, we found the cumulative incidence of neurologic death in high, moderate, and low risk groups was 49.0%, 20.3% and 0% after 82.5 months (when the last observed neurologic death occurred), *p* < 0.001 (Fig. 1), with 3-year overall survival (OS) rates of 35.3%, 52.8%, and 59.1%, *p* = 0.047 (log-rank test), respectively.

**Fig. 2:**
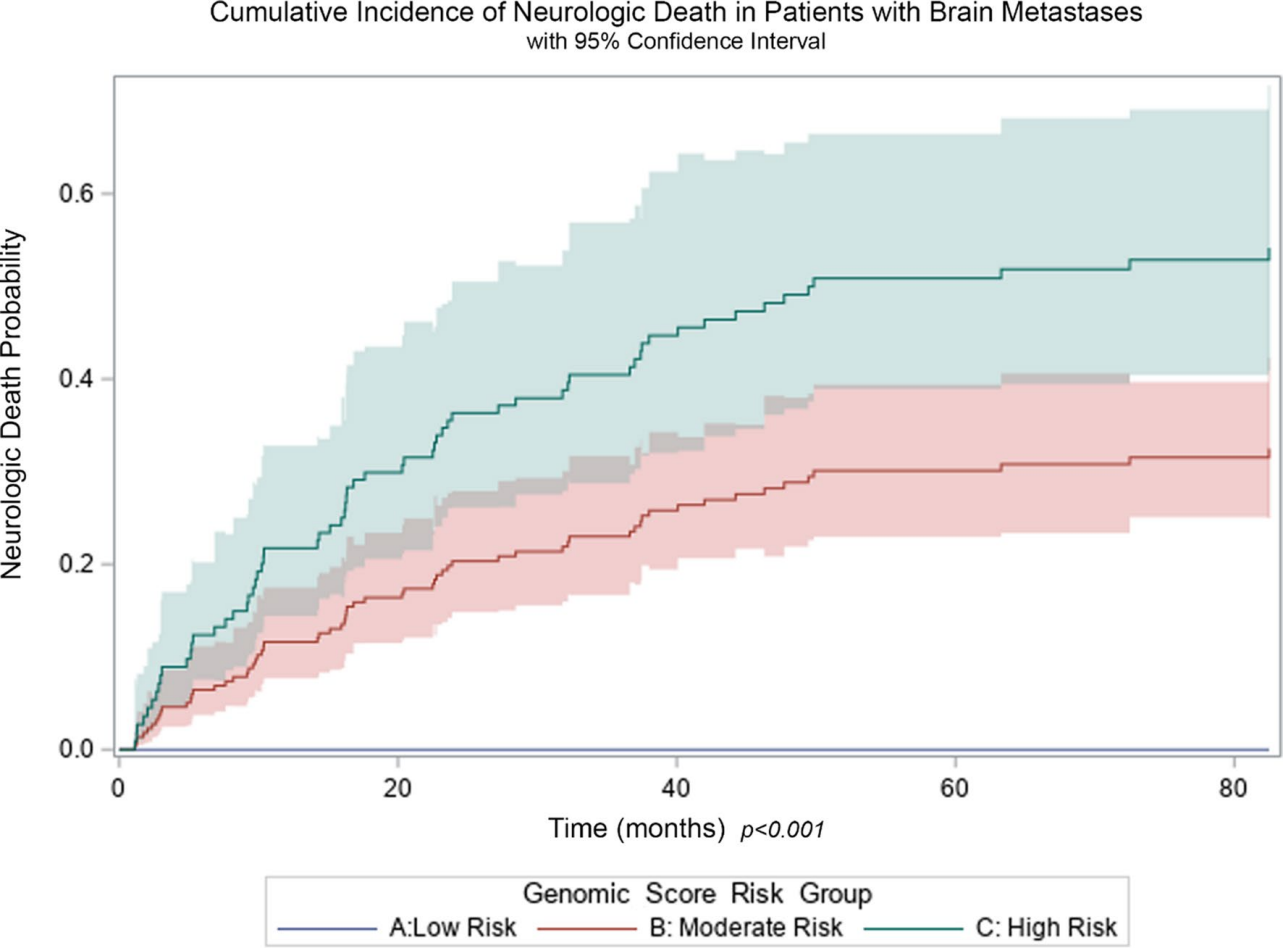
Probability of neurologic death among patients with brain metastases for genomic score risk groups, low risk (blue), moderate risk (red) and high risk (green) *p* < 0.001

Similar to the entire cohort, in the subset of patients who developed brain metastases, the genomic risk score was significantly associated with the risk of neurologic death with a HR of 2.87 (95% CI 2.09–3.94, *p* < 0.001). The cumulative incidence of neurologic death in the high, moderate, and low risk groups was 52.4%, 30.4%, and 0% after 82.5 months (when the last observed neurologic death occurred), *p* < 0.001 (Fig. 2), with 3-year OS rates of 30.6%, 36.4%, and 40.4%, *p* = 0.29, respectively.

**Fig. 3:**
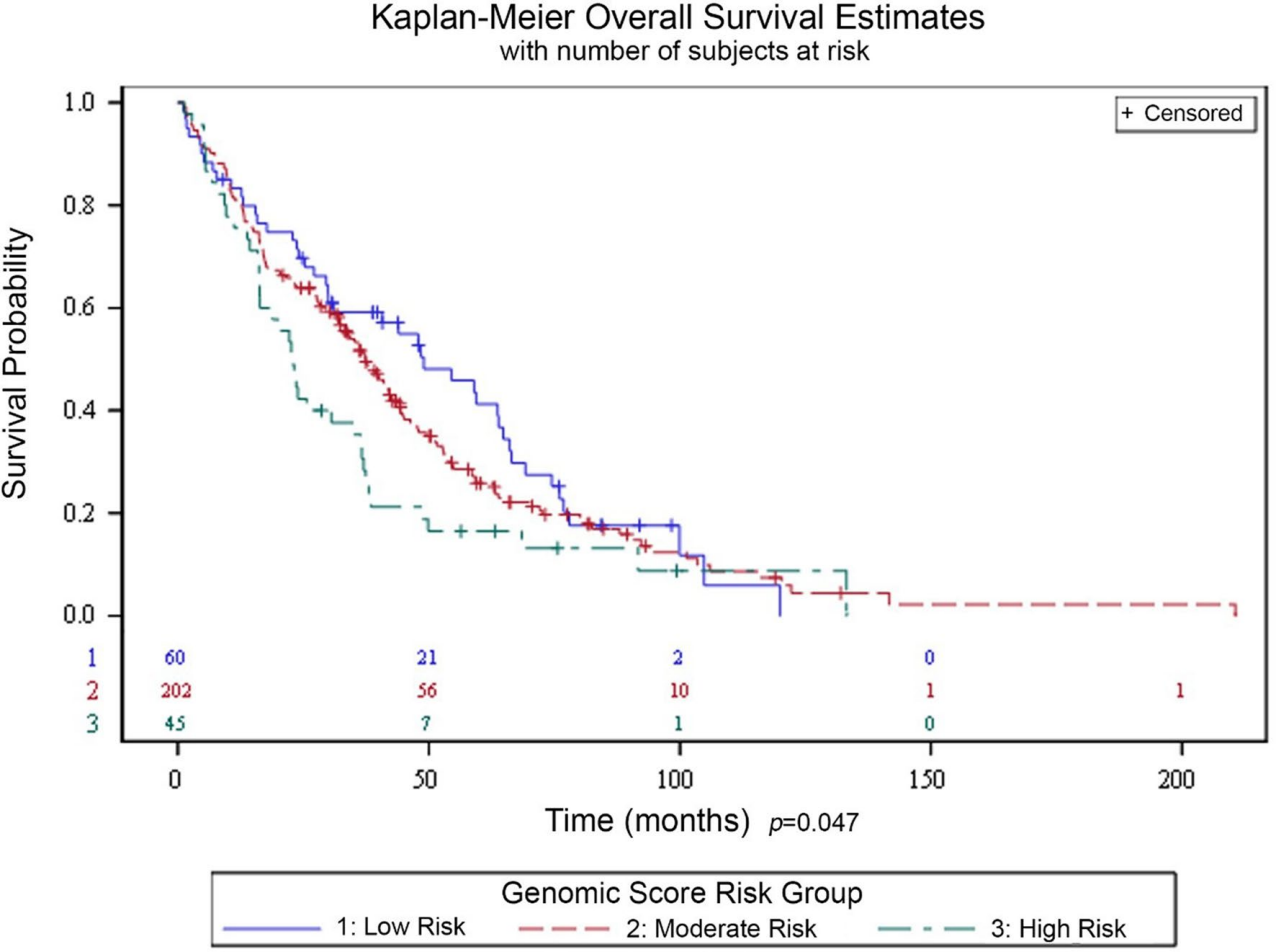
Kaplan-Meier estimates of overall survival by genomic score risk groups, low risk (blue), moderate risk (red) and high risk (green) *p* = 0.047

In additional exploratory analyses, the genetic risk score was associated with OS, with significant differences in survival across the three risk groups, *p* = 0.047 (log-rank test; Fig. 3). When stratified by patient subgroups both with and without targetable mutations (*EGFR*, *ROS1*, *BRAF*, *NTRK3*and *ALK*), the genetic risk score remained significantly associated with neurologic death. While a distinct separation was seen between all three risk groups in the patients with no targetable mutations, this distinction was observed only between the low-risk group and the combined moderate- and high-risk groups in the subgroup with one or more targetable mutations. Finally, the genetic risk score remained an independent predictor of neurologic death when adjusting for patient characteristics, including KPS, number of brain metastases, overall disease burden (none, oligo-metastatic, widespread), and type of brain irradiation (WBRT, GKRS, both, or none), *p* < 0.001 (Wald test).

## Discussion

Comprehensive genomic profiling has been used recently as a tool for risk stratification of patients with metastatic cancer. In its early forms, CGP was predominantly used to identify actionable mutations for which patients could be triaged to systemic therapies targeting these specific driver mutations. Successes of CGP in NSCLC include treatment of *EGFR*, *ALK*, *ROS1*, *RET*, *KRAS* and *BRAF*-mutated cancers with specific small molecule inhibitors [18].

Glynn et al. published a study demonstrating that CGP done at time of first metastasis in NSCLC could be used to risk-stratify patients based on their risk of developing brain metastases [13]. Abdulhaleem et al. published a study demonstrating that CGP could also be used to identify the risk of brain metastasis phenotypes, such as large symptomatic lesions, small numerous lesions, and metastasis subtypes that would rapidly re-seed the brain [14]. These proof-of-principle studies, demonstrating that brain metastasis phenotype is largely influenced by genetics, led our team to hypothesize that brain metastases leading to neurologic death was likely another phenotype with a genetic foundation.

Neurologic death from brain metastasis can be caused by several events, including local failure of resistant metastases, unrelenting re-seeding of the brain, hemorrhage of metastases, leptomeningeal dissemination, and long-term neurologic sequela from the original presentation of the brain metastasis. Prior preclinical investigations have demonstrated that certain genetic variants are more radioresistant [19] or more likely to lead to leptomeningeal invasion [20]. Some metastases are more likely to become hemorrhagic, strongly suggesting a genetic causation of this phenotype as well [21]. A seminal publication by Brastianos et al. demonstrated that specific mutations were more likely to be found in brain metastases when evaluating multiple primary cancer types, suggesting that these genetic changes were instrumental in the cancer types more readily seeding the brain [22].

In spite of the several mechanisms leading to neurologic death, the implication of likely neurologic death may be similar. Brain metastases can have a heterogeneous presentation depending on how early or late in the natural history they develop. Patients who develop brain metastases as an end-stage event have a low risk of neurologic death due to competing risk of death from other causes; these patients may not benefit from aggressive CNS-directed therapy. The QUARTZ study showed that in NSCLC patients with poor performance status, there was no benefit from treating brain metastases with whole brain radiotherapy (WBRT) [23]. Those who have a higher risk of neurologic death, either due to well-controlled extracranial disease or more poorly controlled intracranial disease, may benefit from more aggressive CNS-directed therapy [24]. Those with a lower risk of neurologic death may benefit from de-escalation, delayed treatment, or no treatment at all to avoid treatment related toxicities [25].

Biomarkers for brain metastasis behavior have traditionally been challenging to find due to the heterogeneity of the brain metastasis population [26]. However, it has long been known that NSCLC patients with driver mutations *EGFR* and *ALK* had a greater risk of developing brain metastases and developed them earlier in their natural history than their wild-type counterparts [27]. In this study, neither of these mutations was more common in the high-risk group than in the lower or moderate ones, and in fact the incidence of *EGFR* mutations was lower in the high-risk group. This may be due to improved outcomes with the escalation of therapy with blood-brain barrier penetrant agents for patients harboring these mutations, in addition to traditional local therapy approaches [28].

A potential use for biomarkers is the optimization of medical decision-making. Radiation oncologists have been among the first to attempt to use CGP for risk stratification in order to triage patients to the most efficient radiation treatment. Choi et al. demonstrated that the likelihood of oligometastatic versus widespread failure could be risk stratified in NSCLC using liquid-biopsy based CGP [29]. The predictive value of CGP was highly statistically significant and the findings were later validated by an independent dataset, thus suggesting that this risk stratification score could be used to recommend patients with risk of oligometastatic failure undergo ablative radiation [30].

Biomarker-based decision making for the radiotherapeutic management of brain metastases may have positive downstream effects on the cost of management. A recent study by Shenker et al. found that the decision that most affected cost of care for brain metastasis patients is the upfront decision of stereotactic radiosurgery vs. WBRT [31]. Prior analyses have demonstrated that nomogram use may decrease the cost of management in brain metastasis patients [32]. Genetic biomarkers are more refined than traditional nomograms, and as such they may offer greater potential in cost reduction and improved outcomes [33]. At the present time, biomarker discovery for brain metastases is focusing on tumor tissue biomarkers [34], circulating tumor cells and DNA, as well as extracellular vesicles [35]. The advantage of CGP acquired by liquid-biopsy in the present study is its non-invasiveness, commercial availability, and CLIA-certified standards.

There are several limitations to the present dataset. As a retrospective analysis, several biases could exist, including patient selection bias. Validation with an independent dataset would be needed prior to this risk stratification system becoming operationalized clinically for triage of patients. The rapid pace of the development of CNS-penetrant systemic therapy agents is also a limitation, as new agents targeted towards mutations in the signature may impact its prognostic value. However, in spite of the hypothesis-generating nature of retrospective datasets, this study serves as an important proof of concept that CGP can risk-stratify NSCLC patients as a whole, and among those with brain metastases with regard to their likelihood of dying from neurologic causes without requiring invasive biopsy.

## Conclusions

The development of a liquid-biopsy based genomic signature to predict neurologic death in NSCLC patients appears feasible. If properly validated, the risk stratification system may serve as a basis for clinical trial design for triaging NSCLC patients to escalated versus de-intensified CNS-directed management, such as more frequent surveillance imaging, radiation dose escalation, or use of CNS-penetrating systemic therapies.

## Data Availability

The data presented in this study are available on request from the corresponding author due to privacy and legal restrictions.
